# Transcriptomic, metabolomic, and ATAC-seq analysis reveal the regulatory mechanism of senescence of post-harvest tomato fruit

**DOI:** 10.3389/fpls.2023.1142913

**Published:** 2023-03-08

**Authors:** Susu Guo, Yanhai Ji, Yanyan Zheng, Christopher B. Watkins, Lili Ma, Qing Wang, Hao Liang, Chunmei Bai, Anzhen Fu, Ling Li, Demei Meng, Mingchi Liu, Jinhua Zuo

**Affiliations:** ^1^ State Key Laboratory of Food Nutrition and Safety, College of Food Science and Engineering, Tianjin University of Science & Technology, Tianjin, China; ^2^ Key Laboratory of Vegetable Postharvest Processing, Ministry of Agriculture, Institute of Agri-Products Processing and Food Nutrition, Beijing Academy of Agriculture and Forestry Sciences, Beijing, China; ^3^ Beijing Key Laboratory of Fruits and Vegetable Storage and Processing, Institute of Agri-Products Processing and Food Nutrition, Beijing Academy of Agriculture and Forestry Sciences, Beijing, China; ^4^ Key Laboratory of Biology and Genetic Improvement of Horticultural Crops (North China) of Ministry of Agriculture, Institute of Agri-Products Processing and Food Nutrition, Beijing Academy of Agriculture and Forestry Sciences, Beijing, China; ^5^ Key Laboratory of Urban Agriculture (North) of Ministry of Agriculture, Beijing Vegetable Research Center, Institute of Agri-Products Processing and Food Nutrition, Beijing Academy of Agriculture and Forestry Sciences, Beijing, China; ^6^ School of Integrative Plant Science, Horticulture Section, College of Agriculture and Life Science, Cornell University, NY, Ithaca, United States; ^7^ College of Food Science and Biotechnology, Tianjin Agricultural University, Tianjin, China

**Keywords:** storage, tomato, transcriptome, metabolome, ATAC-seq, transcription factor

## Abstract

Several physiological changes occur during fruit storage, which include the regulation of genes, metabolisms and transcription factors. In this study, we compared ‘JF308’ (a normal tomato cultivar) and ‘YS006’ (a storable tomato cultivar) to determine the difference in accumulated metabolites, gene expression, and accessible chromatin regions through metabolome, transcriptome, and ATAC-seq analysis. A total of 1006 metabolites were identified in two cultivars. During storage time, sugars, alcohols and flavonoids were found to be more abundant in ‘YS006’ compared to ‘JF308’ on day 7, 14, and 21, respectively. Differentially expressed genes, which involved in starch and sucrose biosynthesis were observed higher in ‘YS006’. ‘YS006’ had lower expression levels of CesA (cellulose synthase), *PL* (*pectate lyase*), *EXPA* (*expansin*) and *XTH* (*xyglucan endoglutransglucosylase/hydrolase*) than ‘JF308’. The results showed that phenylpropanoid pathway, carbohydrate metabolism and cell wall metabolism play important roles in prolonging the shelf life of tomato (*Solanum lycopersicum*) fruit. The ATAC-seq analysis revealed that the most significantly up-regulated transcription factors during storage were TCP 2,3,4,5, and 24 in ‘YS006’ compared to ‘JF308’ on day 21. This information on the molecular regulatory mechanisms and metabolic pathways of post-harvest quality changes in tomato fruit provides a theoretical foundation for slowing post-harvest decay and loss, and has theoretical importance and application value in breeding for longer shelf life cultivars.

## 1 Introduction

Fruits undergo a series of physiological changes after harvest, such as changes in color, firmness, nutrient contents, and flavor, which involve a series of metabolic processes involving sugars and acids (Li et al., 2020; You and Van Kan, 2021). Flavonoids, which constitute an important group of phenolic secondary metabolites, have a wide range of biological activities, including anti-allergic, anti-bacterial, anti-cancer and antioxidant activity (Takshak and Agrawal, 2019; El-Bilawy et al., 2022). Tomato have been characterized as an essential dietary flavonoids source because of a high consumption worldwide and rich in flavonoids (Wang et al., 2022). In tomato fruit, the main flavonoids have been identified to be rutin, naringenin, and chalconatingenin (Slimestad et al., 2008; Safaie Farahani and Taghavi, 2018). Flavonoids are derived from the phenylpropanoid pathway (Dong and Lin, 2021).

Tomato flavor characteristics are caused by the relative contents of acids and sugars (Xiao et al., 2018; Zhang et al., 2018). The activities of sucrose synthase (SUS), and sucrose-phosphate synthase (SPS) are essential for sugar accumulation in fruit. Neutral invertase (NI) also contributes to fruit sweetness by catalyzing the degradation of sucrose into glucose and fructose (Schaffer and Petreikov, 1997; Giovannoni et al., 2017). Studies have shown that the use of ethanol treatment can delay the peak of soluble sugar content and increase the sugar-acid ratio of tomatoes there by maintainng good flavor (Yanuriati et al., 1999). Organic acids, which constitute the foundation of amino acids, vitamins, flavonoids, and aromatic substances synthesis, are mainly derived from the conversion of sugars and products related to cell wall metabolism (Pech et al., 2012). The most prevalent organic acids in tomato fruit are citrate, malate, and oxalic acid. The malate synthesis and degradation pathways within glycolysis and the tricarboxylic acid cycle are well known. During postharvest storage, the content of sugar and titratable acid always increases, and the higher the content of sugar and acid, the more conductive to the flavor of tomato fruit (Kanayama, 2017). Studies have shown that the increase of total sugar and the decrease of acidity will shorten the shelf life of cherry tomatoes (Li et al., 2017).

Fruit softening results from cell wall disruption caused by structural changes, hydration of cell wall polymers and dozens of cell wall-related enzymes involved in the biological processes (Saladie et al., 2007). Some enzymes have been identified specific functions involving in altering cell wall structure, limiting the expression of a single gene does not necessarily affect softening (Zhang et al., 2017; Cai et al., 2022). The most studied cell wall modification-related enzyme is polygalacturonase (PG). Some studies showed its activity was inhibited with transgenic tomatoes also showing soften phenotype, which suggested PG activities alone inadequate affect fruit softening and ripening (Cooley and Yoder, 1998; Rao and Paran, 2003). Additional cell wall modification-related enzymes include pectinesterase (PE), pectate lyase (PL) (Torun and Uluisik, 2022), β-galactose (β-GAL) (Bai et al., 2021a), xyloglucan transglycosylase/hydrolase (XTH) (Morales-Quintana et al., 2020) and expansin (EXP) (Wang et al., 2021; Veronico et al., 2022). These enzymes play roles in cell wall degradation in coordinating with fruit ripening and senescence.

In addition to the transcriptome and metabolome, transposase accessible chromatin also involved in regulating fruit ripening processes. It has been reported that the sorghum and rice genomes have accessible chromatin regions (ACRs) (Wilkins et al., 2016; Zhou et al., 2021). However, researchers have not showed that there are patterns of chromatin accessibility in tomato fruit. Furthermore, the association between chromatin accessibility and gene expression has not been thoroughly explored during senescence of tomato fruit. The regulatory ‘YS006’, is a tomato cultivar with longer shelf lift than ‘JF308’. To explore the underlying regulation of the long shelf-life of ‘YS006’ and the molecular mechanism of fruit senescence, we performed a comprehensive transcriptome analysis of the ‘JF308’ and ‘YS006’ during storage, as well as assays for transposase accessible chromatin sequencing (ATAC-seq) and metabolomic analysis. We identified important differential expressed genes (DEGs), differential accumulated metabolites (DAMs), and transcription factors (TFs) associated with maintenance of quality during storage. These results can construct a metabolic regulatory network.

## 2 Material and methods

### 2.1 Plant material and growth conditions

The high-sugar tomato cultivars ‘YS006’ and ‘JF308’ were planted on a commercial vegetable farm located in Tongzhou district, Beijing, China. Red ripe fruit were harvested and subsequently stored at 25 °C and 80% relative humidity. Fourty-five fruits with uniform size were sampled after days 7, 14, and 21 of storage, and named J7, J14, and J21 for ‘JF308’ and Y7, Y14, and Y21 for ‘YS006’. Each whole fruit was washed, dried and sliced into small pieces, after which the pieces were mixed together, frozen in liquid nitrogen, and then stored at -80 °C until used.

### 2.2 Soluble sugars and titratable acids analysis

Soluble sugar and titratable acids contents were measured as described previously by Freschi et al. (2010) and Yaman and Bayoιndιrlι (2002), respectively.

### 2.3 Transcriptome analysis

#### 2.3.1 Transcriptome sequencing

Eighteen libraries representing the six tomato samples and three replicates were constructed for RNA sequencing (RNA-seq). The RNA concentration and purity were measured by a NanoDrop 2000 instrument (Thermo Fisher Scientific, Wilmington, DE). RNA integrity was determined through the RNA Nano 6000 Assay Kit of the Agilent Bioanalyzer 2100 system (Agilent Technologies, CA, USA). The specific steps are described in previous studies published by our team (Ma et al., 2021; Fu et al., 2022).

#### 2.3.2 Transcriptome statistical analysis

Reads with ideal suitability or mispairing were explained based on the reference genome. HISAT2 tools was used to map the reads to the reference genome. Screening and functional annotations of DEGs were performed as previously described (Ma et al., 2020; Fu et al., 2021)

### 2.4 Metabolomic analysis

#### 2.4.1 Metabolite extraction

A 100 μL sample was picked and positioned in an Eppendorf tube, extracted with 300 μL of methanol, 20 μL internal standard (2-chloro-L-phenylalanine) added, after centrifugation at 10249.1 g for 15 min at 4 °C. The supernatant (200 μL) was transferred into a fresh 2 mL LC/MS glass vial. 200 μL supernatant was used for the UHPLC-QTOF-MS analysis (Bai et al., 2021b).

#### 2.4.2 Data preprocessing and annotation

MS raw data files were transformed to the mzXML format using Proteo-Wizard, and processed *via* the R package XCMS. The preprocessing results were used to generate a data matrix that consisted of the retention time (RT), mass-to-charge ratio (m/z) values, and peak intensity. The R package CAMERA was used for annotations after XCMC data processing. An in-house MS2 database was applied to identify the metabolite.

### 2.5 ATAC-seq

#### 2.5.1 Experimental method of ATAC-seq

The nucleus was extracted by gradient centrifugation, and the DNA was purified using the Qiagen PCR (28006) purification kit. A total of 50000 cells were centrifuged at 500x g for 5 min at 4 °C, and the supernatants was removed. The cells were washed with cold PBS once, and the centrifugation was repeated. The cells were then suspended in cold lysis buffer, and the supernatant was removed after centrifugation. The transposing reaction system was configured for use with Tn5 transposase. The cell nuclei were suspended in the transposing reaction mixture, and the DNA was purified after incubating at 37 °C for 30 min. PCR mixtures included the purified DNA, and PCR was subsequently performed. The final DNA libraries were run on an Illumina platform after the DNA was purified (Cai et al., 2022).

#### 2.5.2 ATAC-seq data preprocessing and annotation

The raw Illumina sequencing reads were processed to remove any adapters. Bowtie2 software was used to identify high-quality reads. DeepTools v2.07 was applied to map the density distribution of the sequencing reads, and the results were visualized as heatmaps. For detection of genome-wide peak regions, MACS2 v2.1.1 software was used. Peak regions associated with a false discovery rate (FDR)< 0.05 were ultimately selected. Using BLAST, we compared the sequences of the different peak-associated genes enriched in the promoter region with the sequence information available in the nonredundant (Nr), SwissProt, Gene Ontology (GO), Kyoto Encyclopedia of Genes and Genomes (KEGG), Clusters of Orthologous Groups of proteins (COG), EuKaryotic Orthologous Groups (KOG), evolutionary genealogy of genes: Non-supervised Orthologous Groups (EggNOG), and Pfam databases to identify genes for subsequent gene functional analysis (Zhou et al., 2021).

## 3 Results

### 3.1 Soluble sugar contents and titratable acidity

**Figure 1:**
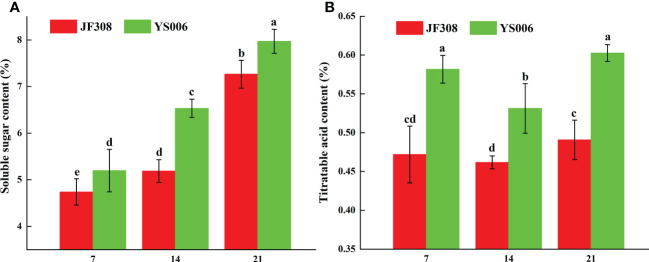
Soluble sugar content **(A)** and titratable acid **(B)** in ‘JF308’ and ‘YS006’ tomato fruit during storage. The means (± SD, n = 4) are shown. Different letters indicate differences at *P* < 0.05.

The relative contents of sugars and acids determine the flavor of tomato fruits, we analyzed these compounds in the ‘JF308’ and ‘YS006’ tomato fruit. The soluble sugar content in ‘JF308’ and ‘YS006’ increased over time. The Y21 fruit had the highest content, and the soluble sugar content in ‘JF308’ and ‘YS006’ ranged from 4.73 to 7.26%, and 5.19 to 7.97%, respectively (**Figure 1A**). The titratable acid content in ‘JF308’ and ‘YS006’ decreased and then increased during storage time. The titratable acidity of ‘JF308’ and ‘YS006’ ranged from 0.46 to 0.49%, and from 0.53 to 0.60%, respectively. Significant changes in titratable acid content were detected in ‘JF308’ and ‘YS006’ at day 7, day 14 and day 21 (**Figure 1B**). Similar trends with soluble sugar, titratable acid synthesis related genes and metabolites were also observed in metabolome and transcriptome analysis.

### 3.2 Metabolomic analysis of the fruit of the tomato cultivars

**Figure 2:**
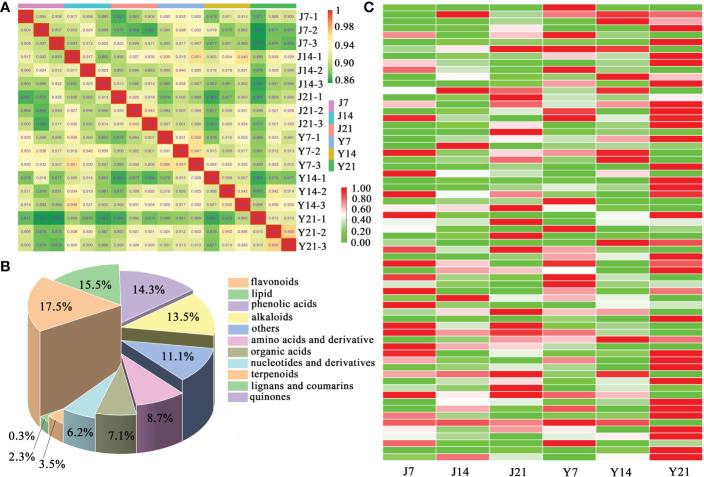
Data assess of metabolomic analysis. **(A)** Sample-to-sample correlation analysis. The Spearman rank correlation is displayed as an evaluation index of biological replicated correlations. The closer R^2^ to 1, the stronger correlation between two replicates. **(B)** Type and proportions of 1006 metabolites. **(C)** Hierarchical clustering of whole metabolites in the tomato fruit ‘JF308’ and ‘YS006’. The Y-axis represents different metabolites, and the X-axis represents six samples.

Sample-to-sample correlation analysis of the metabolites from the fruit of the two cultivars was performed (**Figure 2A**). The correlation was more than 0.9 between each fruit sample. In total, 1006 metabolites were found, including 88 amino acids and derivatives, 71 organic acids, 176 flavonoids, 156 lipids, 62 nucleotides and derivatives, and 71 phenolic acids (**Figure 2B**). A heatmap cluster analysis was performed to illustrate the differences among the 1006 metabolites of the two cultivars. The results of this analysis showed ‘YS006’ storage for 21 days accumulated more metabolites than ‘JF308’ storage for 21 days (**Figure 2C**).

#### 3.2.1 Differentially accumulated metabolite (DAM) analysis

**Figure 3:**
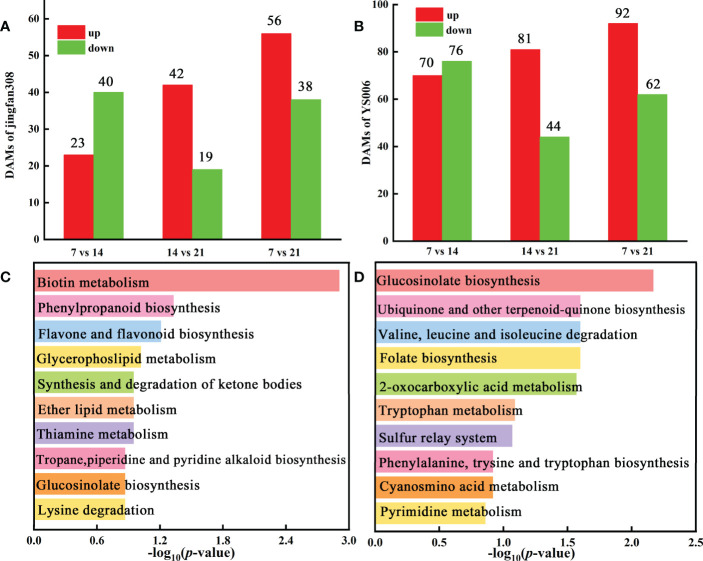
Identification and functional characterization of DAMs in ‘JF308’ and ‘YS006’ during post-harvest storage. **(A)** Number of DAMs in ‘JF308’ fruit. The red color represents the number of increases; the green shows the number of decreases, X-axis represents the three J7 vs. J14, J14 vs. J21, and J7 vs. J21 comparison groups. **(B)** Number of DAMs in ‘YS006’, the red columns indicate the number of increases; the green columns indicate the number of decreases, and the X-axis represents Y7 vs. Y14, Y14 vs. Y21, Y7 vs. Y21 comparison groups. **(C)** KEGG enrichment analysis of the DAMs in the J7 vs. J21 comparison group. **(D)** KEGG enrichment analysis of the DAMs in the Y7 vs. Y21 comparison group.

Adhering to our screening conditions (variable importance in projection (VIP) > 1, fold change (FC) > 1, and *P* < 0.05), we analyzed metabolite changes in ‘JF308’ during storage. Altogether 23 metabolites increased, and 40 metabolites decreased in the J7 vs. J14 group. In the J14 vs. J21 comparison group, 42 and 19 DAMs increased and decreased, respectively (**Figure 3A**). KEGG enrichment analysis of the DAMs revealed phenylpropanoid biosynthesis, synthesis and degradation of ketone bodies, and flavone and flavanol biosynthesis were the most abundant (**Figure 3C**).

**Figure 4:**
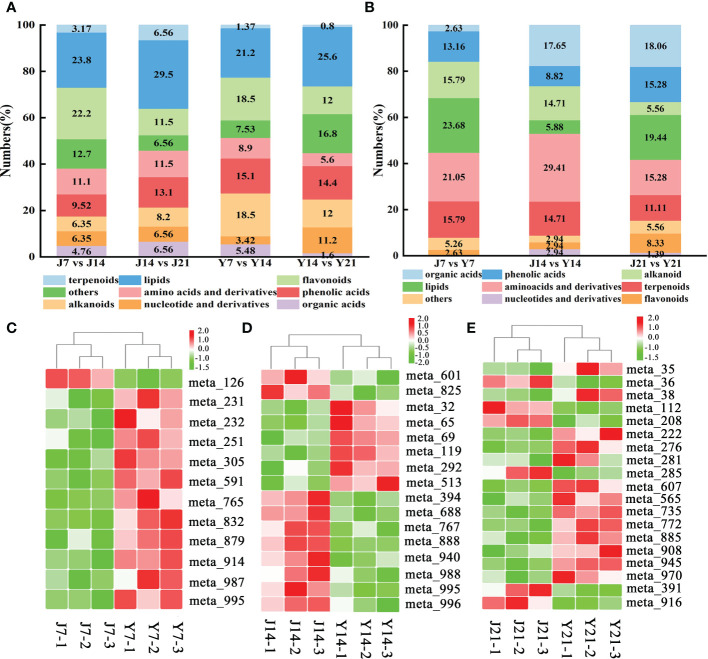
Type, proportion of DAMs and differential accumulated sugars, acids, and flavonoids in different comparison groups. **(A)** Type and proportion of DAMs in J7 vs. J14, J14 vs. 21, Y7 vs. Y14, and Y14 vs. Y21 comparison groups. The horizontal axis represents the groups of the four comparison groups, and the vertical axis represents the percentage of total metabolites. **(B)** Type and proportion of DAMs in the J7 vs. Y7, J14 vs. Y14, J21 vs. Y21 comparison groups. The horizontal axis represents the groups of the four comparison groups, and the vertical axis represents the percentage of total metabolites. **(C)** Differentially accumulated sugars, acids, and flavonoids in the J7 vs. Y7 comparison group. The red color represents increase, the green color represents decrease, J7-1 represents the day 7 replicate of ‘JF308’, and Y-axis represents the metabolite number. **(D)** Differentially accumulated sugars, acids, and flavonoids in the J14 vs. Y14 comparison group. **(E)** Differentially accumulated sugars, acids, and flavonoids in the J21 vs. Y21 comparison group.

A total of 146, 125 DAMs were found in the two comparative groups (Y7 vs. Y14, Y14 vs. Y21), with 70 up-regulated and 76 down-regulated in Y7 vs. Y14. (**Figure 3B**). The DAMs were mapped to KEGG metabolic pathways. Four pathways were identified with the storage time for ‘YS006’: 2-oxocarboxylic acid metabolism, valine, leucine and isoleucine degradation, ubiquinone and another terpenoid-quinone biosynthesis, and phenylalanine, tyrsine and tryptophan biosynthesis (**Figure 3D**). Metabolites that differentially accumulated between Y7 and Y14 fruit were evaluated, which included 31 lipids, 27 alkaloids, 27 flavonoids, 23 phenolic acids, 13 amino acids and derivatives, 8 organic acids, and other compounds (**Figure 4A**). 81 DAMs were increased and 44 DAMs were decreased in Y14 vs. Y21 fruit, these DAMs included 32 lipids, 19 others, 18 phenolic acids, 15 flavonoids, 15 alkaloids, 14 nucleotides and derivatives, and seven amino acids and derivatives (**Figure 4B**).

#### 3.2.2 DAMs related to sugars, acids, and flavonoids

Six saccharides and alcohols, namely D-arabinono-1,4 lactone, l-glucose, D-fructose, 3-phospho-D-glyceric acid, D-saccharic acid, and D-fructose-1,6-biphosphate were identified. All the saccharides increased in Y7 fruit compared to J7 fruit. Among the saccharides, D-fructose-1,6-biphosphate was 1.90-fold higher in Y7 than in J7. Most flavonoids (rhodiolgin, quercetin-3-o-(6’-acetyl) galactoside, quercetin-7-o-(6’-malonyl) glucoside, quercetin-3-o-neohesperidoside, quercetin-3-o-sophoroside-7-o-rhamnoside, quercetin-3-o-(6’-feruloyl) glucoside-7-o-rutinosine) present a 1.38-3.52 increase in Y7 fruit compared with J7 fruit; however, the contents of calycosin-7-o-glucoside, and chrysoeriol-5-o-glucoside decreased by 0.52 and 0.46-fold, respectively (**Figure 4C**; **Appendix 1**). KEGG enrichment analysis of the DAMs revealed fructose and mannose metabolism, glycolysis/gluconeogenesis, and galactose metabolism were the most abundant.

The contents of amino acids and their derivative including l-valine, trans-4-hydroxy-l-proline, l-isoleucine, l-lysine, l-tryptophan, and γ-glutamyltyrosine were 1.07-1.93-fold higher in Y14 than in J14. The flavonoids had lower contents in Y14 fruit than in J14 fruit; these compounds included narigenin (5,7,4’-trihydroxyflavanone), apigenin-7-o-glucoside, 6-hydroxykaempferol-7-glucoside, naringenin-7-o-neohesperidoside, quercetin-3,7-di-o-glucoside, kaempferol-6,8-di-c-glucoside-7-o-glucoside, quercetin-3-o-(6’-feruloyl) glucoside-7-o-rutinoside, and luteolin-7-o-(2’-o-rhamnosyl) sophoroside-5-o-glucoside (**Figure 4D**; **Appendix 2**).

The organics acids, methylmalonic acid, acetoxyacetic acid, aminomalonic acid, valproic acid, cis-aconitic acid, 2-isopropylmalic acid, D-galacturonic acid, gluconic acid, D-erythrose-4-phosphate, and D-maltose differentially accumulated. In Y21, the content of methylmalonic acid increased by 2.07-fold in ‘YS006’ compared to ‘JF308’. In addition, the content of 2-isopropylmalic acid was 2.78 -fold higher in Y21 than in J21. Additionally, the contents of most saccharides and alcohols were 1.36-1.51-fold higher in Y21 than in J21; However, this was not the case for D-erythrose-4-phosphate, whose content was markedly lower by 0.62-fold in ‘YS006’ compared to ‘JF308’. Flavonoids were the second most abundant metabolites that differentially accumulated between the two cultivars. Among these flavonoids, tricin (5,7,4-trihydroxy-3’,5’-dimethoxyflavone), dihydromarein, isorhamnetin-3-o-gallate, kaempferol-3-o-sambubioside, quercetin-3-o-(2’-o-rhamnosyl) galactoside, hesperetin-5,7-di-o-glucoside, kaempferol-3-o-(2-o-xylosyl-6-o-rhamnosyl) glucoside contents were 1.2-2.6-fold higher in Y21 than in J21. In addition, baicalein, acacetin, 5-hydroxy-3,7,4’-trimethoxyflavone, and quercetin-3-o-robinobioside were 0.18-0.7-fold more abundant in Y21 than in J21 (**Figure 4E**, **Appendix 3**). KEGG analysis indicated that DAMs were involved in the amino acid biosynthesis, cyanoamino acid metabolism, 2-oxocarboxylic acid metabolism, and carbon metabolism, demonstrating that flavonoids, organic acids, saccharides, and alcohols influence fruit quality to some extent.

### 3.3 Transcriptome profiling in ‘JF308’ and ‘YS006’ tomato fruit during post-harvest storage

For ‘JF308’ tomato fruit, a total of 3352 (1462 up- and 1890 down-regulated) and 2035 (991 up-and 1044 down-regulated) genes were differentially expressed in the pairwise comparisons of J7 and J14, J14 and J21, respectively (**Supplementary Figure 1A, C**; **Appendix 8**). The top enriched KEGG pathways enriched in these DEGs were amino acids biosynthesis, carotenoid biosynthesis, phenylalanine, tyrosine and tryptophan biosynthesis, ubiquinone and another terpenoid-quinone biosynthesis, and terpenoid backbone biosynthesis (**Supplementary Figure 1B, D**; **Appendix 8**). With respect to the GO terms, 2676 genes were noted as being involved in biological processes, 2822 unigenes were involved with cellular components, and 1794 were involved in molecular functions, including carbohydrate metabolic processes, the cell wall, and xyloglucan: xyloglucosyl transferase activity (**Supplementary Figure 2A**; **Appendix 8**).

In total, 4065 and 2286 genes were consistently differentially expressed between Y7 and Y14 fruit, and between Y14 and Y21 fruit, respectively. There were 1711 DEGs up-regulated and 1395 down-regulated between Y7 and Y14. 2354 DEGs were up-regulated and 891 were down-regulated, between Y14 and Y21. GO term enrichment identified genes were enriched in the isoprenoid biosynthetic process, responses to chitin, cell walls, ammonia-lyase activity, UDP-glucose 4-epimerase activity, and xyloglucan: xyloglucosyl transferase activity (**Supplementary Figure 2B**; **Appendix 8**). KEGG pathway enrichment analysis was also utilized to identify the metabolic pathways of the DEGs. DEGs that participated in plant hormone signal transduction, terpenoid backbone biosynthesis, and phenylalanine metabolism were enriched in the ‘YS006’ tomato fruit.

#### 3.3.1 Expression of cell wall modification-related genes

Cell wall-related DEGs were selected, and the expression levels in the fruits of the cultivars were compared. Modest decreases in cell-wall related gene expression were observed in J21 vs. Y21 fruit, the results are consistent with the longer shelf life of ‘YS006’ tomato fruit. The relative expression levels of 5 key genes were compared: *β-galactosidase 5* (*β-GAL5*), *cellulose synthase* (*CESA*), *expansin* (*EXPA*), *pectate lyase* (*PL*), *xyloglucan endotransglucosylase/hydrolase* (*XTH9*). The expressions levels of *β-GAL5* in the J7 and Y7, and J21 and Y21 comparison group were all increased, except in J14 and Y14, which had a down-regulated trend. The log_2_FC of *β-GAL5* was 0.88 in J21 and Y21 fruit, -0.86 in J14 and Y14 fruit, and 0.66 in J14 and Y14 fruit. The expression of *CESA* gradually decreased during storage. The log_2_FC of J7 and Y7 fruit was 3.67, whereas that of J21 and Y21 was -1.17. The expression of *EXPA* tended to be down-regulated with increasing storage time; the log_2_FC of J7 and Y7 was -0.91, and that of J21 and Y21 was -0.97. The expression of another gene, *PL*, was also strongly down-regulated over storage time, and the log_2_FC of J7 and Y7 fruit was -0.65, and that of J14 and Y14 reached -0.70. At day 21, the expression of *XTH* in ‘YS006’ was lower than it was in ‘JF308’ fruit; however, on day 7, this gene was up-regulated in ‘JF308’ compared with ‘YS006’ (**Appendix 4–6**).

#### 3.3.2 Expression of phenylpropanoid and flavonoid biosynthesis-related genes in two cultivars fruit

**Figure 5:**
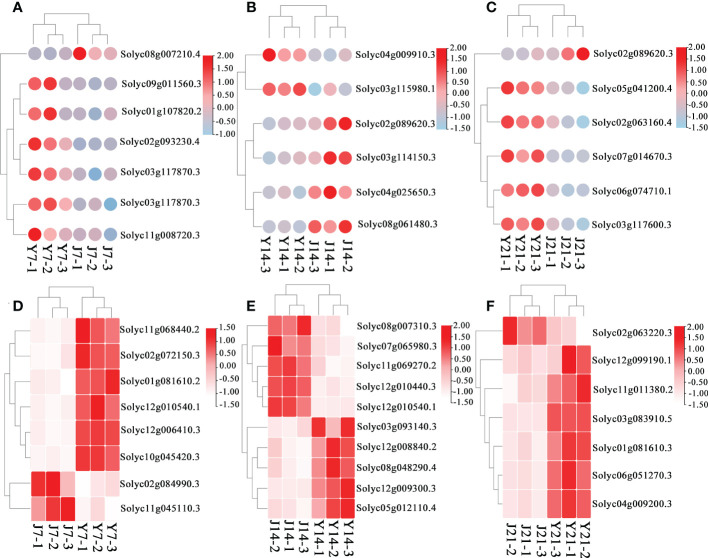
DEGs in the J7 vs. Y7, J14 vs. Y14, and J21 vs. Y21 comparison groups. **(A)** DEGs related to flavonoid metabolism in the J7 vs. Y7 comparison group. The red color represents upregulation, and the white color represents downregulation. **(B)** DEGs related to flavonoid metabolism in the J14 vs. Y14 comparison group. **(C)** DEGs related to flavonoid metabolism in the J21 vs. Y21 comparison group. **(D)** DEGs related to sucrose metabolism in the J7 vs. Y7 comparison group. The red color represents upregulation, and the blue color represents downregulation. **(E)** DEGs related to sucrose metabolism in the J14 vs. Y14 comparison group. **(F)** DEGs related to sucrose metabolism in the J21 vs. Y21 comparison group.

DEGs associated with the phenylpropanoid and flavonoid pathways were investigated in all comparison groups. In addition to cultivar differences, the genes involved in the flavonoid and phenylpropanoid pathways at day 7 were expressed more than those at day 21 both in ‘JF308’ and ‘YS006’ tomato. Transcripts of the phenylpropanoid pathway were increased in Y7 fruit relative to J7 fruit; this was the case for transcripts of the genes encoding *scopoletin glucosyltransferase*, *caffeoyl-CoA o-methyltransferase*, *4-coumarate-CoA ligase* which were increased. (**Figure 5A**; **Appendix 4**). In the J14 and Y14 comparison group, the expression of *UDP-glycosyltransferase 89b2*, *stearoyl-desaturase 6*, and *aldehyde dehydrogenase* was down-regulated (**Figure 5B**; **Appendix 5**). The ‘YS006’ fruit on day 21 exhibited a high expression anthocyanidin reductase, which may lead to accumulation of flavonoids (**Figure 5C**; **Appendix 6**).

#### 3.3.3 Expression of genes related to carbohydrate metabolism

Transcriptomics analysis of ‘JF308’ and ‘YS006’ provide an opportunity to map how genotype can affect carbohydrate metabolism-related pathways during storage. On day 7, the increased expression of *glucan endo-1,3-β-glucosidase*, *UDP-glucuronate 4-epimerase* (*GAE1*), *α-α-trehalose-phosphate* (*TPS*), *o-fucosyltransferase 20 isoform X1* (*OFUT20*), and *xyloglucan 6-xylosyltransferase 2* (*XXT2*) in both ‘JF308’ and ‘YS006’ fruit indicated that the expression genes involved in carbohydrate transport and metabolism increased (**Figure 5D**; **Appendix 4**). On day 14 the increase expression of *sucrose synthase* (*SUS*), a reversible enzyme that synthesizes and decomposes sucrose, is generally believed to play a primary role in decomposing sucrose, explaining that the content of sucrose decreased, which is consistent with the metabolomic results. The expression of *galacturonosyltransferase 8* (*GAUT8*), *o-fucosyltransferase 6* (*OFUT6*), *mannose-6-phosphate isomerase 2* (*PMI2*), which involved in fructose and mannose metabolism and starch and sucrose metabolism, also increased (**Figure 5E**; **Appendix 5**). The expression of *mannose-1-phosphate guanylyltransferase 1* (*CYT1*), *hexosaminidase 2* (*HEXO2*), *acid β-fructofuranosidase* (*TIV1*), and *vacuolar inhibitor of fructosidase 1* (*VIF1*) which involved in the pentose phosphate pathway, and starch and sucrose metabolism were up-regulated at day 21 for both cultivars (**Figure 5F**; **Appendix 6**).

### 3.4 Association analysis of genes and metabolites related to fruit flavor

**Figure 6:**
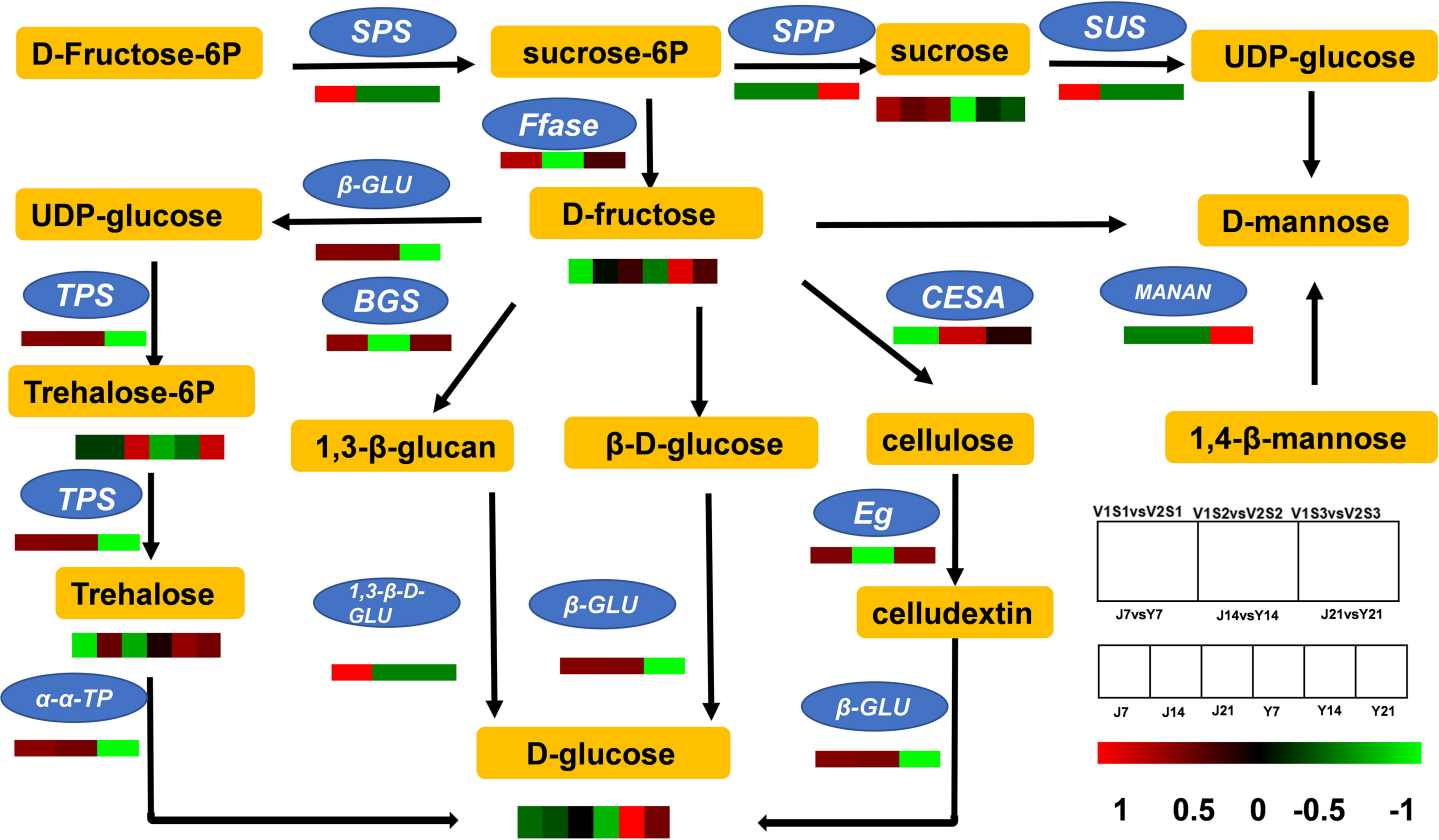
Pathways of primary carbohydrate metabolism and sugar accumulation in tomato. Comparisons of DEG expression levels in the J7 vs. Y7, J14 vs. Y14, and J21 vs. Y21 comparison groups. The colors represent the different multiples between groups, and the metabolites are expressed as J7, J14, J21, Y7, Y14, and Y21. SPS, sucrose-phosphate synthase; SUS, sucrose synthase; Ffase, β-fructofuranosidase; β-GLU, β-glucosidase; TPS, trehalose 6-phosphate synthase; α-α-TP, α-α-trehalose phosphate; 1,3-β-D-GLU, 1,3-β-glucan synthase; CESA, cellulose synthase; Eg, endoglucanase; MANAN, mannan-endo-1,4-β-mannosidase.

The DEGs and DAMs in ‘JF308’ and ‘YS006’ were mapped to their corresponding KEGG pathway to determine the relationship between vital genes and metabolites associated with fruit aroma. We identified 12 DEGs, and five metabolites were found to participate in the starch and sucrose pathways (**Figure 6**). *Glucan endo-1,3-β-D-glucosidase* was up-regulated by 2.03-fold in Y21 fruit compared with J21 fruit. As a result, the glucose content increased in the ‘YS006’ fruit. The expression of *cellulose synthase* and *endoglucanase* decrease by 1.77-fold and 0.88-fold, respectively, in Y21 fruit. *SUS* is expressed in the cytoplasm, and mainly catalyzes UDP and sucrose to form fructose and UDPG (Tao et al., 2021; Sun et al., 2022). The expression of *SUS* was up-regulated by 1.09-, 1.03-, and 1.03-fold in the J7 and Y7, J14 and Y14, and J21 and Y21 comparison groups, respectively. This corresponds to the decrease in sucrose content in post-harvest fruit identified *via* the metabolomics analysis. *β*-fructofuranosidase expression was up-regulated by 1.08-fold in the J21 and Y21 comparison group, leading to an increased abundance of D-fructose.

### 3.5 ACRs in ‘JF308’ and ‘YS006’ tomato fruit during post-harvest storage

#### 3.5.1 Accessible chromatin landscape of tomato fruit

**Figure 7:**
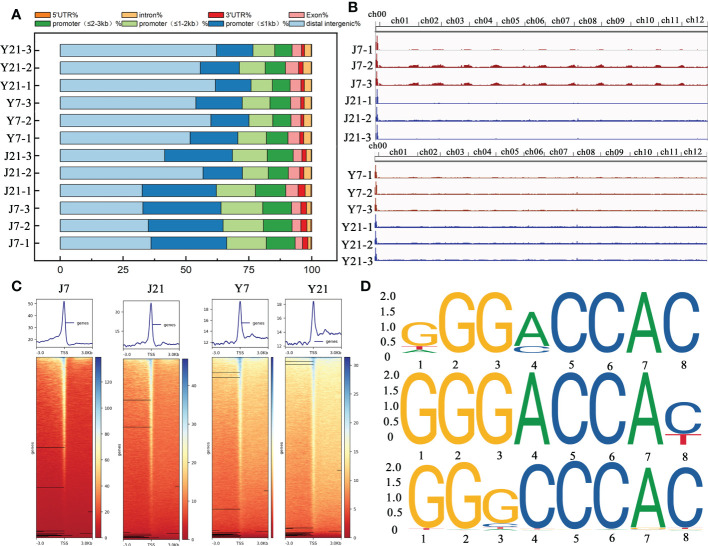
Overall ATAC-seq results of tomato fruit. **(A)** Genomic distributions of all the ACRs (accessible chromatin regions) found among the ATAC-seq data. The X-axis shows the percentage, and the Y-axis indicates the samples. **(B)** Genome browser view of ATAC reads collected at the J7, J21, Y7, and Y21 stages. **(C)** ACR density plots and heatmaps of over-enriched ACRs at the J7, J21, Y7, and Y21 stages. **(D)** Enriched motifs of *TCP4*, *TCP5*, and *TCP24* in the J21 vs. Y21 comparison group. The larger the letter is, the greater the probability that the nucleotide or amino acid is present at that location, (expressed as bits).

To explore the accessible chromatin alterations under different storage times, ‘JF308’ and ‘YS006’ tomato fruits were collected for ATAC-seq. In *Arabidopsis*, ACRs are mainly enriched in the upstream transcription start site (TSS) (Maher et al., 2018; Sijacic et al., 2018). Most ACRs in tomato fruit were located in distal intergenic regions (35.02% ~ 62.13%) or with in the promoter (≤ 1 kb) (14.49% ~ 31.08%). Approximately 8.64% ~ 16.62% of the ACRs were located within 1-2 kb of the promoter, and only 1.15% ~ 2.8% were located within the 3’UTR (**Figure 7A**). According to the results of ATAC-seq analysis, the ACRs of ‘JF308’ and ‘YS006’ were found to be differentially enriched at day 7 and 21 after storage (**Figure 7B**). We subsequently examined the signs of these ACRs located in the TSSs (**Figure 7C**). The data indicated that the enriched ACRs on days 7 and 21 showed that the strongest signal was concentrated near the TSS.

#### 3.5.2 KEGG annotation of differentially accessible regions

We performed a KEGG analysis to identify the pathways associated with DARs. The DARs of J7 and Y7 fruit are predominantly enriched in genes involved with plant hormone signal transduction, amino acid biosynthesis, carbon metabolism, and the circadian rhythm. The DARs of J21 and Y21 fruit were enriched with those involved in plant hormone signal transduction, and MAPK signaling pathway, cysteine and methionine metabolism and sphingolipid metabolism. We then performed a GO functional analysis of J7 and Y7 DARs, and for the cellular component terms, the endoplasmic reticulum membrane, and Golgi membranes were enriched. Similarly, the molecular function category terms that included DNA-binding transcription activity were more abundant in J7 than in Y7. According to the GO annotation, the DARs in genes associated with biological process categories, including the response to oxygen-containing compounds, were enriched in J21fruit compared with Y21 fruit. Additionally, in terms of cellular component category, the nucleus, and cell wall were enriched. Moreover, genes associated with molecular function were more highly enriched in DNA-binding TF activity and sequence-specific DNA binding.

#### 3.5.3 Enriched motif results and description of TFs in answer to senescence

We identified 24151 positively and 42493 negatively enriched DARs between J7 and Y7 fruit. For the J21 and Y21 contrast, 130 positively enriched and 6140 negatively enriched DARs were identified, respectively. These DARs were subjected to motif analysis, and 60 and 88 unique motifs were identified in J7 compared with Y7, and J21 compared with Y21, respectively. Through alignment of the sequences with those in a plant TF (motif) database, *TCP2*, *TCP3*, *TCP4*, *TCP5*, and *TCP24* were recognized, showing that TCPs may play essential roles in the senescence of tomato fruit during storage (**Figure 7D**). Furthermore, several TFs were classified as members of C2H2 zinc finger basic region-leucine zipper factors (bZIP) and basic helix-loop-helix factors (bHLH) TF families, these TFs were identified as candidates controlling intracellular responses to storage (**Appendix 7**). Among the 88 TFs, 33 belong to the bHLH family.

#### 3.5.4 Combined results of ATAC-Seq and RNA-Seq analysis

**Figure 8:**
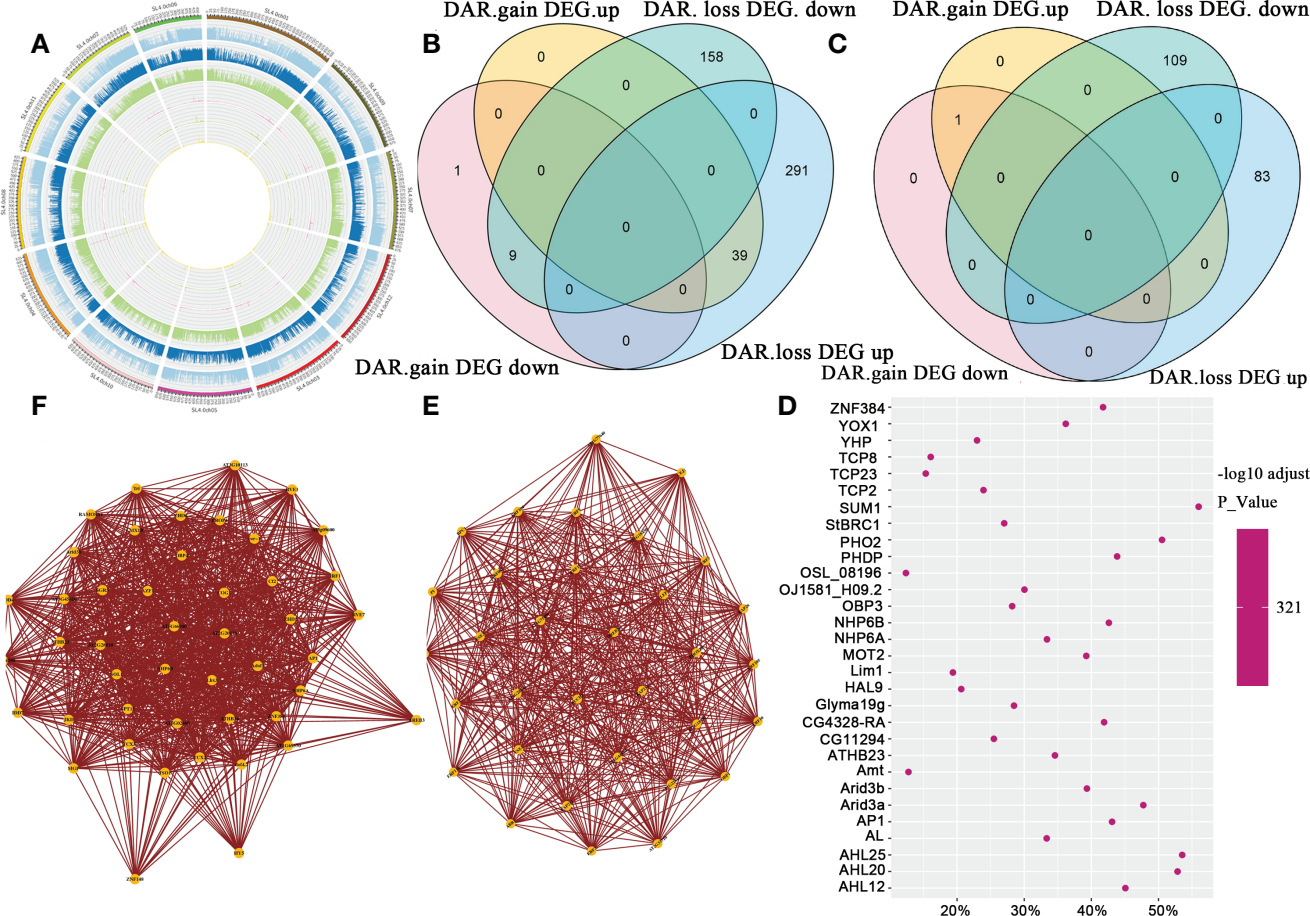
Joint analysis of ATAC-seq and RNA-seq. **(A)** The circus map of ATAC-seq signal and gene expression at the chromosome level. The ATAC-seq signal level on the outer reaction genome and the expression level of genes in the corresponding region of the inner reaction of J7. **(B)** The Venn diagram shows the overlaps between differentially expressed genes (DEGs), differentially accessible region (DAR)-related genes in a comparison of J7 and Y7. **(C)** Venn diagram shows the overlaps between DEG and DAR-related genes in comparing J21 and Y21. **(D)** Enrichment statistics of transcription factors in differential open regions the point is these enriched transcription factors, and the side is the synergistic interaction of transcription factors. **(E)** Transcription factor interaction diagram. **(F)** The X-axis is the proportion of enrichment transcription factors in the open region and the Y-axis is the transcription factor; the color in the figure is the size of the corrected p-value.

To demonstrate the association of ATAC-Seq signals and gene expression levels at the chromosome level, we created Circos plots for each group of samples. The ATAC-Seq signal value was high, consistent with genes with high expression levels in J7 fruit (**Figure 8A**). To explore the correlation between chromatin openness and gene expression levels, we divided the genes into five levels. We counted the gene sets showing differences in ATAC-seq DAR genes (the genes represented by the TSS closest to the DAR center) and expression levels; these genes could be divided into four categories: open region gain expression up-regulated, open region gain expression down-regulated, open region loss expression up-regulated, and open region loss expression down-regulated. The quantitative relationships of the four categories in J7 fruit compared to Y7 fruit are shown in **Figure 8B**. In contrast with Y7, there were 369 up-regulated and 177 down-regulated DEGs. In addition, 49 increased DARs and 497 decreased DARs were identified. Similarly, **Figure 8C** shows the quantitative relationships of the four categories in J21 contrast with Y21. We first counted the TFs that regulate both DARs and DEGs, and then scanned the locations of these TF sets in the DARs. We used AME software to determine the enrichment of TFs in different open areas: AHL12, ZNF384, and SUM1 were the most enriched TFs (**Figure 8D**). At the same time, TFs acting on the same target genes can tend to co-regulation, indicating that they can interact with each other (**Figures 8E, F**). Three pathways were most enriched in J21 and Y21 comparison group: plant hormone signal transduction, amino sugar and nucleotide sugar metabolism, and starch and sucrose metabolism.

## 4 Discussion

Fruit senescence involves a series of very complex physiological changes. The expression of senescence-related genes changes during fruit senescence, and fruit quality change. In tomato fruit, this process is mainly accompanied by quantitative changes in sugars, acids, phenols, and minerals (Kader, 2008). The soluble sugar content in tomato fruit is an important index of fruit senescence and quality deterioration (Gautier et al., 2008; Beckles, 2012). In ‘JF308’ and ‘YS006, the sugar content increases with fruit ripening and senescence. Using a widely-targeted metabolism approach, we found that L-glucose, D-fructose, D-fructose-1,6-biphosphate contents increased in the fruit of both cultivars. The change trend of these sugars is similar to the change trend of fructose and glucose in peach fruit (Cirilli et al., 2016). The combined transcriptomic and metabolomic analysis showed that among the genes involved in starch and sucrose metabolism, the main genes were *SPS*, *TPS*, *SPP*, *SUS* and *β-Glu*. During 7 d of storage, the expression of the *SUS*, *SPS*, *TPS* and *α-α-TP* genes in ‘YS006’ was up-regulated. Moreover, the expression of *β-GLU*, *TPS*, and *α-α-TP* was up-regulated at day 14 d, and *SPP*, *Eg* was up-regulated at day 21. These genes contribute to the synthesis of D-fructose, sucrose, D-glucose, and D-mannose, which also explained why the sugar content in ‘YS006’ is higher than that in the ‘JF308’. A previous study showed that exogenous auxin inhibited the activities of SPS and SUS; these inhibited activities resulted in a decreased sucrose content during tomato fruit ripening (Tao et al., 2022). Our results also showed as increased expression of *SPS* and *SUS* in ‘YS006’, the content of sugar increased, which is consistent with previous study.

During post-harvest storage of fruit, respiration is the main way through which biological activities are maintained. We detected that the methylmalonic acid, 2-isopropylmalic acid in the Y21 was higher than that in the J21 fruit. Studies have shown that the contents of citric acid, malic acid, cis-aconitic acid, succinic acid and other organic acids in cherry tomato fruit decreased at room temperature after harvest, but the contents of these acids increased at low temperature (Tang et al., 2020). Some study also showed UV-C treatment significantly increased the organic acids through up-regulating organic biosynthesis related gene (Yan et al., 2021). Organic acids are the main respiratory compounds in the early stage of storage and respiration is gradually enhanced during post-harvest storage. The content of organic acids in ‘YS006’ was higher than that in ‘JF308’, which may be achieved by slowing down respiratory metabolism.

Flavonoids are naturally occurring compounds in plant tissues and have a variety of functions, including exerting strong antioxidant capacity, protecting against UV-B radiation, protecting against pathogen attack, and acting as signaling compounds that initiate symbiotic relationships (Hichri et al., 2011; Czemmel et al., 2012). Phenylpropanoid metabolism is the main component of secondary metabolism in fruit and vegetables; this process regulates the accumulation of main antibacterial substances such as phenols, flavonoids and lignin, and plays an important role in disease resistance (Liu et al., 2015; Dong and Lin, 2021). In this experiment, compared with those in the ‘JF308’, the expression of genes related to flavonoid synthesis in the ‘YS006’ increased on days 7 and 21, which promoted the accumulation of flavonoids. This result was similar to those found in studies on UV-C irradiation, and exogenous ABA in tomato fruit (Liu et al., 2018; Tao et al., 2020). Our results showed that ‘YS006’ fruit have improved antioxidant capacity through phenylpropanoid metabolism, thereby prolonging the shelf life.

**Figure 9:**
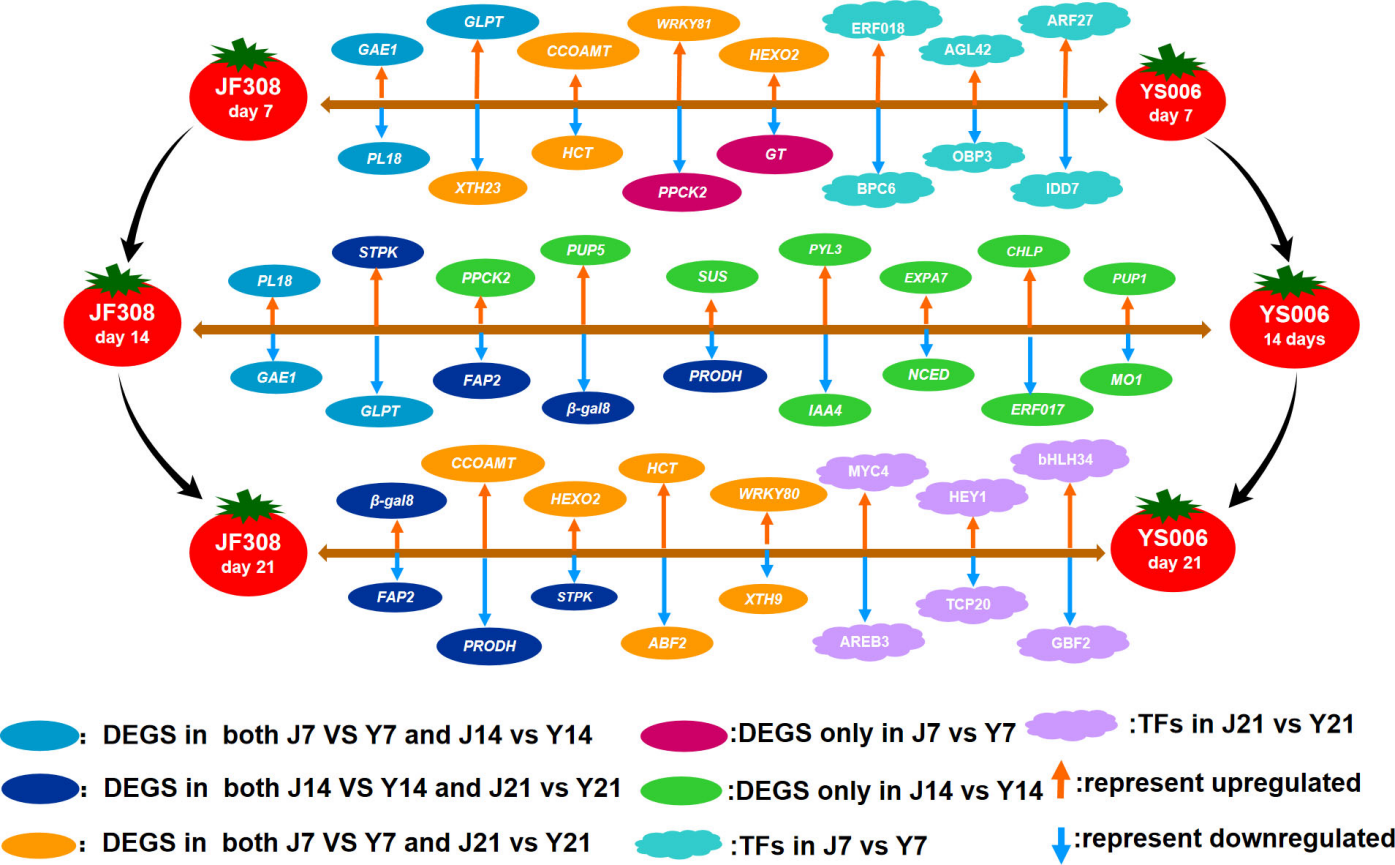
Regulation model of ‘JF308’ and ‘YS006’ tomato fruit during post-harvest storage. The model shows DEGs and TFs that affect fruit quality in the J7 vs. Y7, J14 vs. Y14, and J21 vs. Y21 comparison groups. The oval shapes represent DEGs, and the cloud shapes represent different TFs. The genes are expressed in italics. GAE1, UDP-glucuronate 4-epimerase; PL18. pectate lyase 18; XTH23, xyloglucan endotransglucosyl/hydrolase protein 23; CCOAOMT, caffeoyl-CoA-methytransferase; HCT, shikimate O-hydroxycinnamoyltransferase; PPCK2, phosphoenolpyruvate carboxylase kinase; HEOX2, beta-hexosminidase 2; GT, glycosyltransferase; FAP2, fatty-acid binding protein 2 isoform X3; PUP5, purine permease 5; SUS, sucrose-phosphate synthase; β-gal8, beta-galactosidase; PRODH, proline dehydrogenase; PYL3, abscisic acid receptor; IAA4, auxin-responsive protein IAA4; EXPA, expansin-A7; NCED, 9-cis-epoxycarotenoid dioxygenase; PUP1, purine permease 1; MO1, monooxygenase 1; GHLP, geranylgeranyl diphosphate reductase; ERF017, ethylene responsive factor 017;.

The effect of cell wall-related genes on the senescence of fruit has been thoroughly analyzed (Forlani et al., 2019; Posé et al., 2019). There is scientific evidence that fruit senescence is accompanied by cell wall depolymerization (Shi et al., 2019; Kim et al., 2022). Changes in elemental texture are complex and contribute to cell wall remodeling and changes in cell adhesion and turgor (Zhai et al., 2018; Shi et al., 2021). This effect could stem from the increase in enzymatic activities involved in texture changes, for instance, those of products encoded by *β-Gal*, *CESA*, *PL*, *EXPA*, and *XTH*. Recently extracorporeal enzymology data showed that CesA can promote cellulose accumulation in the presence of UDP-glucose, indicating that no extra factors are needed for *CesA* activity (Purushotham et al., 2016). The expression of *CesA* was higher on day 7 after harvest in ‘YS006’ than ‘JF308’ and down-regulated over time. The levels of *CesA* in the ‘YS006’ were lower than those in the ‘JF308’ on days 14 and 21. In addition, other cell wall-degrading enzymes have been identified, such as PL (Yang et al., 2017; Shi et al., 2022), XTH (Speicher et al., 2018; Ezquer et al., 2020), and EXPA (Mohanty et al., 2018; Ilias et al., 2019), all of which promote cell wall loosening and texture changes. According to the present results, the fruit softening-related genes, *PL*, *EXPA*, and *XTH* all exhibited down-regulated expression in the J14 vs. Y14, and J21 vs. Y21 comparison groups (**Figure 9**). The lower expression of these genes in ‘YS006’ leads to an increase in cell wall soluble pectin and pectin polymerization, which result in prolonged shelf life of ‘YS006’ tomato.

We identified many DARs and found 60 and 88 TFs differentially expressed in J7 compared to Y7, and J21 compared to Y21, respectively. Among the 88 identified TFs, 33 belong to the bHLH family. KEGG analysis showed that plant hormone signal transduction was the most enriched pathway. BHLH TFs compose the most predominant TF gene family in plants and control various biological processes and developments. *SlPRE2*, a bHLH family TF gene, was a negative regulator involved in chlorophyll and carotenoid accumulation, and mediated GA pathway during tomato ripening (Zhu et al., 2017; Zhu et al., 2019). We speculated that in Y21 fruit, genes related to plant hormone signal may be induced through the *bHLH* TFs, thereby delaying the senescence of ‘YS006’ and prolonging their shelf life. *TCP2*, *TCP3*, *TCP4*, *TCP5*, and *TCP24* belong to the bHLH family, which was observed to belong to the CIN subclass (Cubas et al., 1999). Studies have shown that *TCP2* can regulate the development of leaves and dynamic changes in leaf primordium formation (Shleizer et al., 2011). We found that during the process of fruit senescence, the FC of *TCP2*, *TCP3*, *TCP4*, *TCP5*, and *TCP24* expression also increased in the J21 vs. Y21 comparison group. It has been determined that TCP genes may be essential in the management of fruit ripening and senescence.

## 5 Conclusion

The differences in metabolites, genes, and TFs between ‘YS006’ and ‘JF308’ were explored. The results of this study showed that ‘YS006’ accumulated more total sugars, acids, and flavonoids during storage compared with ‘JF308’ fruit. ‘YS006’ with a firmer structure had lower expression levels of *CESA*, *PL*, *EXPA*, and *XTH* than ‘JF308’. The expression of genes associated with phenylpropanoid and flavonoid biosynthesis, increased by day 7 and day 21, which is consistent with the metabolomic data. By using ATAC-seq, we identified 33 TFs belonging to the bHLH family; these TFs, *TCP2*, *TCP3*, *TCP4*, *TCP5*, and *TCP24* are the most significant up-regulated TFs in J21 vs. Y21. The results suggested that the phenylpropanoid pathway, carbohydrate metabolism, and cell wall metabolism function in prolonging the shelf-life of tomato fruit during storage, and provide a theoretical basis for delaying the loss of quality during post-harvest storage.

## Data availability statement

The RNA-seq data have been uploaded successfully in NCBI, accession numbers: SRR23692596, SRR23692595, SRR23692586, SRR23692585, SRR23692584, SRR23692583, SRR23692582, SRR23692581, SRR23692580, SRR23692579, SRR23692594, SRR23692593, SRR23692592, SRR23692591, SRR23692590, SRR23692589, SRR23692588 and SRR23692587. The ATAC-seq data are available from NCBI, accession numbers: SRR23702320, SRR23702319, SRR23702316, SRR23702315, SRR23702314, SRR23702313, SRR23702312, SRR23702311, SRR23702310, SRR23702309, SRR23702318 and SRR23702317.

## Author contributions

ML, JZ and YJ conceived and designed the research. SG, YZ, and DM wrote the article. QW, CB, AF, LM, HL, and LL participated in the related experiments and analyzed the data. CW did a significant revision and give some useful suggestions of the manuscript. All authors contributed to the article and approved the submitted version.
