# Supplementary material for: Transcriptomic, metabolomic, and ATAC-seq analysis reveal the regulatory mechanism of senescence of post-harvest tomato fruit
Source: Front Plant Sci. 2023 Mar 8;14:1142913. doi: 10.3389/fpls.2023.1142913 (PMC10032333; doi:10.3389/fpls.2023.1142913)
Supplement: Supplementary file 1 [file DataSheet_1.docx]

Supplementary Material

**Transcriptomic, metabolomic, and ATAC-seq analyses reveal the regulatory mechanism of senescence of post-harvest tomato fruit**

**Susu Guo^§，1,2^, Yanhai Ji^§，2^, Yanyan Zheng^§，2^, Christopher B. Watkins^3^, Lili Ma^4^, Qing Wang^2^, Hao Liang^2^, Chunmei Bai^2^, Anzhen Fu^2^, Ling Li^4^, Demei Meng^✽,1^, Mingchi Liu ^✽,2^, Jinhua Zuo^✽,2^**

§ These authors contributed equally to this work.

✽Corresponding author, Jinhua Zuo: E-mail address: [zuojinhua@126.com](mailto:zuojinhua@126.com)

**Supplementary Figures**
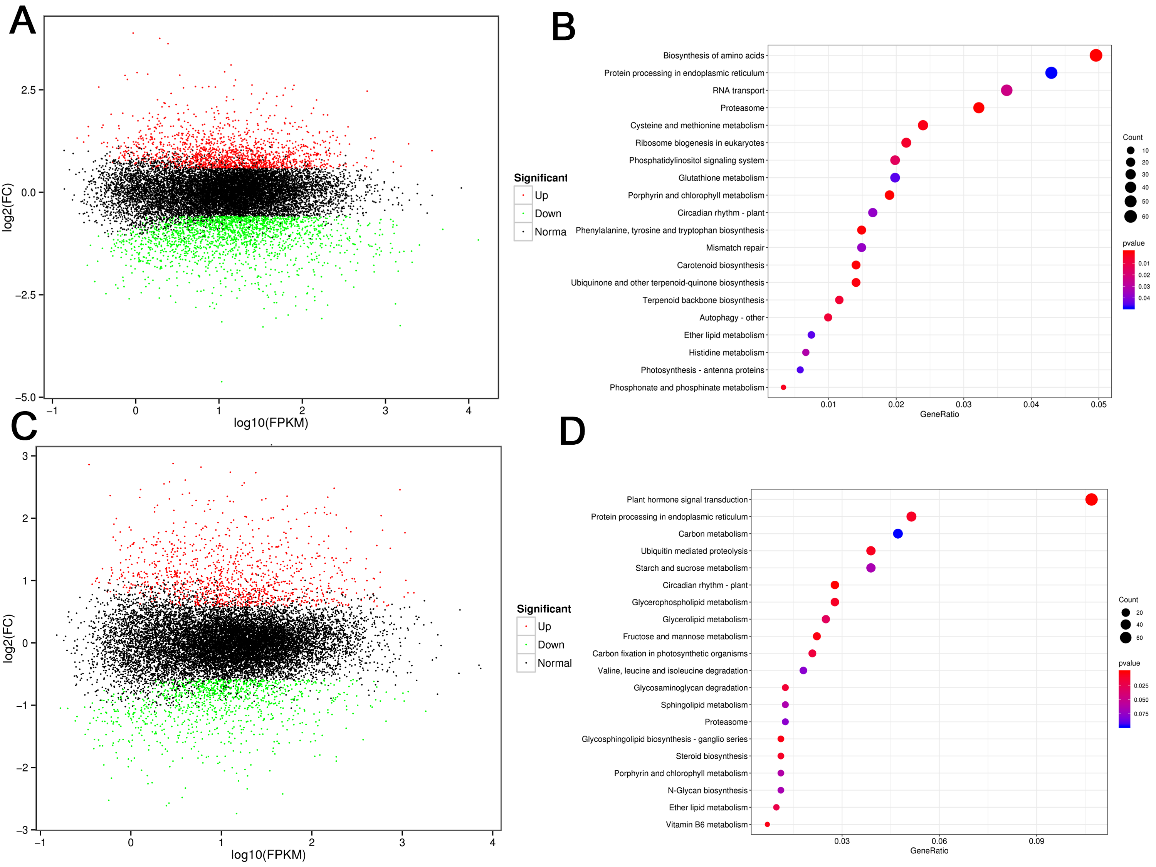


**Supplementary Figure 1.** Numbers and functional annotation of ‘JF308’ fruit. **(A)** The number of J7vs J14. **(B)** KEGG enrichment analysis of the DEGs in the comparisons of J7vs J14. **(C)** The number of J14 vs J21. **(D)** KEGG enrichment analysis of the DEGs in the comparisons of J14 vs J21.


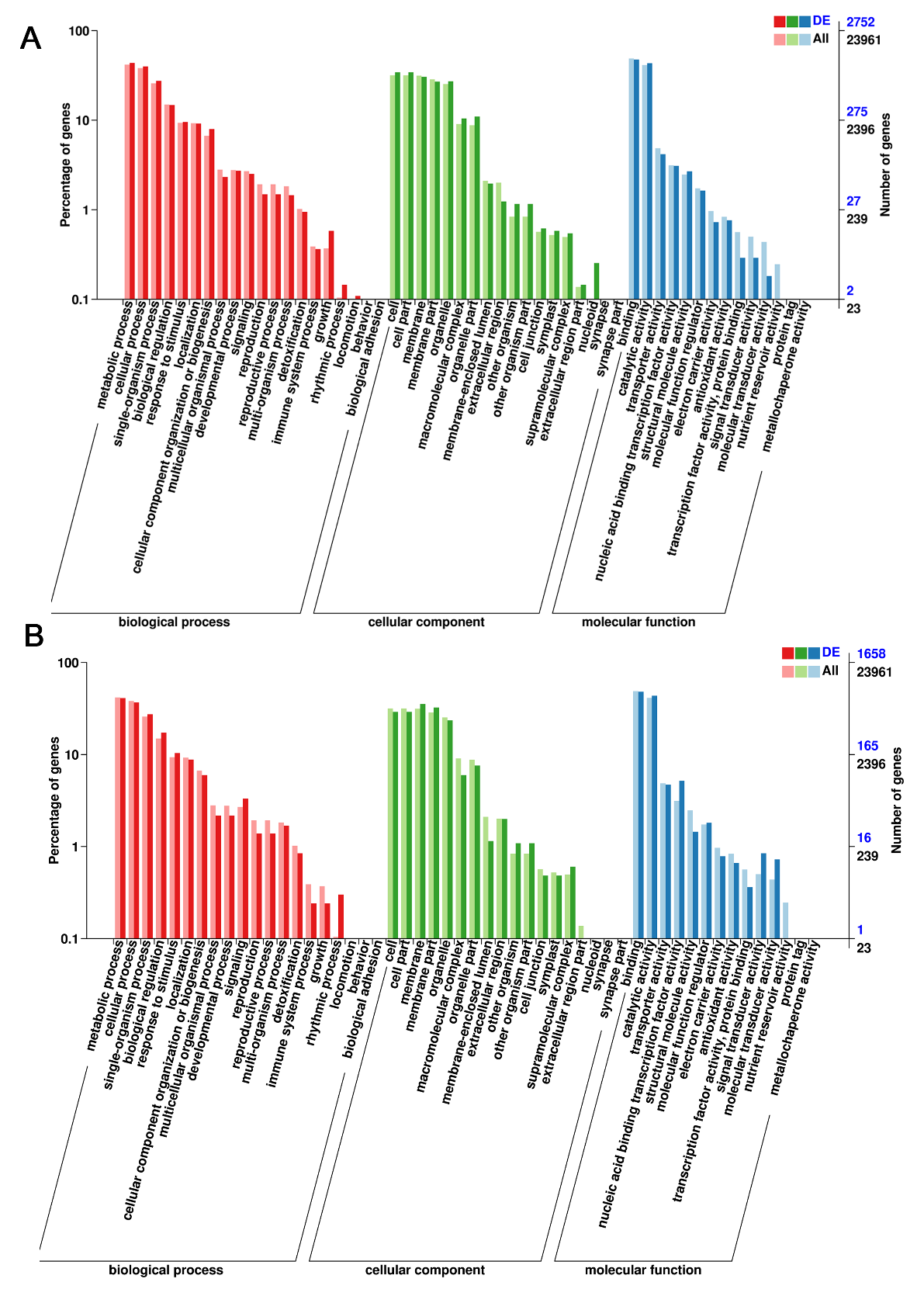


**Supplementary Figure 2.** GO analysis of DEGs in **(A)** ‘JF308’ **(B)** ‘YS006’ and the number and proportion of DEGs in differentially functional categories.
